# Assessment of the awareness and knowledge of cancer survivors regarding the components of metabolic syndrome

**DOI:** 10.1371/journal.pone.0199142

**Published:** 2018-06-19

**Authors:** Yeji Seo, Ji-Su Kim, Eun-shim Park, Eunjung Ryu

**Affiliations:** 1 Student of Graduate school, Chung-Ang University, Seoul, Republic of Korea; 2 Faculty of College of Nursing, Chung-Ang University, Seoul, Republic of Korea; 3 Unit Manager, Cardiovascular Center, Seoul Medical Center, Seoul, Republic of Korea; Laval University, CANADA

## Abstract

This study aimed to assess the prevalence of metabolic syndrome and the level of knowledge and awareness of its related conditions in a sample of cancer survivor patients. In this cross-sectional survey, a self-reported questionnaire was administered to outpatients aged >20 years with a diagnosis of cancer. This self-reported questionnaire on patient demographic characteristics, disease characteristics, and knowledge and awareness of metabolic syndrome was used as an instrument to assess patient’s knowledge of metabolic syndrome. A total of 88 participants were included; of these 34.1% had metabolic syndrome, although only 6.8% of participants were diagnosed with metabolic syndrome. Over half of the participants had heard about metabolic syndrome; however, 70% of the participants did not know about the blood tests for the diagnosis of metabolic syndrome although they were aware of the physical measurements, such as weight, blood pressure, and waist circumference. The highest proportion of correct answers for questions about metabolic syndrome was related to stroke, while the lowest was about cholesterol levels. The proportions of correct answers for selected parameters were as follows: diabetes, 39.1%; adiposity, 47.2%; hypertension, 46.8%; cholesterol levels, 36.7%; arteriosclerosis, 45.5%; myocardial infarction, 37.8%; and stroke, 62.8%. The results suggest that the level of knowledge of metabolic syndrome among the cancer survivors in our sample was poor, although more than one-third of them had metabolic syndrome. Thus, it is essential to educate cancer survivors about metabolic syndrome and its related conditions to improve their overall health and quality of life.

## Introduction

With advances in medical technology, the number of cancer survivors worldwide has markedly increased in recent decades [1]. At present, >25 million people globally have been diagnosed with cancer within the last 5 years [2]. In view of the marked increase in the prevalence of cancer, it is crucial to improving cancer survivorship [2]. Cancer survivorship is a global issue [3] and survivors may encounter several difficulties due to the physical, psychological, and social consequences of cancer and its treatment [1]. Therefore, different intervention strategies to improve survivorship are needed.

Among the constellation of abnormalities that comprise metabolic syndrome (MetS), cardiovascular disease was recently identified as the leading cause of death among cancer survivors [4]. Compared to the general population, cancer survivors have a greater risk of developing secondary diseases such as cardiovascular disease and diabetes, as well as physical deterioration [3]. Therefore, there has been an increased focus on long-term health issues, including MetS, among cancer survivors [5].

According to the most widely-used criteria set by the National Cholesterol Education Program Adult Treatment Panel III (NCEP-ATP III) [6], MetS is defined as the presence of ≥3 of the following 5 components: abdominal obesity, high fasting blood glucose levels, high blood pressure (BP), low serum levels of high-density lipoprotein (HDL) cholesterol, and hypertriglyceridemia [7]. Interestingly, MetS, which is characterized by insulin resistance, dyslipidemia, hypertension, and central obesity, is strongly associated with a higher number of cancer risk factors, including cardiovascular disease, and can be managed by adopting a healthy lifestyle [4,8–10]. Therefore, the role of the healthcare provider is important among cancer survivors in order to manage and educate individuals to maintain self-care. Furthermore, in patients with MetS, it may be more effective to manage the combination of diseases, including hypertension, diabetes, hyperlipidemia, and abdominal obesity, rather than managing these conditions separately [4]. Appropriate management of MetS is important to improve survivorship. Nevertheless, to our knowledge, no specific guidelines are available for the management of MetS among cancer survivors [11].

A previous study showed that the MetS prevalence among cancer survivors ranged from 26% to 55% [11] and that the risk ratio was 2.2 to 4.4 times higher than that among the general population [8]. However, the cause of MetS in cancer survivors remains unclear. A range of risk factors for MetS among cancer survivors has been reported, including local cancer treatment (surgery and radiotherapy), general cancer treatment (chemotherapy and endocrinotherapy), hormonal changes (growth hormone, thyroid hormone, and deficiency of testosterone), metabolic imbalances, sympathetic nervous system changes, and health-related lifestyle choices (physical activity, diet, and smoking) [11–13]. As the causes of MetS among cancer survivors may differ from those in the general population [11,14], it is crucial to develop guidelines that focus on cancer survivors in order to improve survivorship.

To establish guidelines for managing MetS among cancer survivors, it is crucial to first understand the information needed by cancer survivors [15]. Therefore, it is important to assess the awareness and knowledge of MetS among cancer survivors. To our knowledge, this has not been reported to date. In the present study, we aimed to evaluate the level of awareness and knowledge of MetS in order to develop interventions targeting cancer survivor patients at increased risk for MetS to improve their survivorship. Specifically, we sought to identify the prevalence, awareness, and knowledge of MetS and its components among cancer survivors.

## Materials and methods

### Ethics statement

Ethical approval was obtained from the institutional review board of the National Cancer Center (IRB No: 2016–098) prior to the commencement of the study. The purpose of the study was explained to the participants before they completed the questionnaire. The study was conducted after written informed consent was obtained and participants were informed that they could withdraw participation at any point, and that no additional risks or costs were associated with study participation.

### Design and sample

We employed a descriptive design to assess the prevalence of MetS and the level of knowledge and awareness of its related conditions in a sample of cancer survivors. We targeted patients diagnosed with cancer at S Medical Centre. Following surgery or treatment, all of the patients underwent follow-up treatment at the outpatient department. Participants were recruited by research assistants who were trained as per the study protocol.

To avoid recruitment bias, the participants in the hospital were screened according to the outpatient register. Inclusion criteria for participants were as follows: age > 20 years; diagnosis with a primary cancer; no cognitive deficit; provision of informed consent; and ability to communicate and understand the content of the questionnaire and respond to it. Exclusion criteria were as follows: patients with recurrent cancer and serious health conditions or advanced disease; patients were also excluded if they were unable to complete the questionnaire. Research data from 100 patients were collected, and 88 datasets were used in the final analysis; 12 datasets were excluded from the analysis because of insufficient responses.

### Instruments

Data regarding the following demographic characteristics among cancer survivors were obtained: sex, age, marital status, religion, educational status, occupation, and economic status. The cancer-related characteristics collected in this study included the cancer diagnosis, patient age at cancer diagnosis, cancer treatment, and cancer stage. We also evaluated the history and current status of the following variables by reviewing the medical records: hypertension, diabetes, and hypercholesterolemia with relevant medication; waist circumference (WC); systolic and diastolic BP; fasting blood sugar (FBS) levels; and serum biochemistry values.

According to the revised NCEP criteria [6], a diagnosis of MetS requires the presence of ≥3 of the following criteria: 1) WC >90 cm in men and >80 cm in women, according to the International Obesity Task Force criteria for the Asia-Pacific population [16]; 2) triglyceride levels ≥150 mg/dL or medication use; 3) HDL cholesterol levels <40 mg/dL in men and <50 mg/dL in women or medication use; 4) BP ≥130/85 mmHg or antihypertensive medication use; and 5) FBS levels ≥100 mg/dL or medication use (insulin or oral agents).

To assess the awareness of MetS among cancer survivors, we used a modified protocol based on a previous study investigating the awareness of MetS among elderly Korean individuals [17]. The questionnaire included 9 questions regarding the awareness of MetS on the following topics: MetS diagnosis; whether participants had ever heard of MetS, wanted information on MetS, or wanted to consult with their healthcare provider for information on MetS; awareness of their own measurements of WC, FBS level, BP, cholesterol level, and triglyceride level.

The questions related to MetS knowledge used in this study were adapted from a previous study by Becker et al. [18] assessing the knowledge of MetS among college students. The original scale included 90 items across 7 categories. We assessed the validity of this questionnaire for use among cancer survivors among 6 oncologic experts, and then omitted 10 items with an item-level content validity index (I-CVI) of <0.79. Most of the deleted items were related to changes in pregnancy. Therefore, all the MetS knowledge questions exhibited an I-CVI of 0.92 and a scale-level content validity index average of 0.92. Accordingly, a total of 80 questions were used to assess the knowledge of patients regarding the conditions that commonly characterize MetS; the questions were divided into 7 categories: diabetes (13 questions), adiposity (9 questions), hypertension (10 questions), high serum cholesterol levels (6 questions), arteriosclerosis (17 questions), stroke (10 questions), and myocardial infarction (15 questions). The response options to the questions were “true,” “false,” or “do not know,” and patient responses were scored. The “correct” response was awarded 1 point and the “incorrect” and “do not know” responses were awarded 0 points. The maximum possible total score for the MetS questions was 80.

### Data collection

The data for this study were collected between January 9, 2017, and March 31, 2017. The questionnaire was administered to participants after they received outpatient care. Participants completed the questionnaire in a counseling office located adjacent to the outpatient care site; the questionnaire required approximately 20 min for completion. The purpose of the study was explained, and informed consent was obtained from all of the participants by a researcher; participants were then asked to complete the questionnaire individually. Cancer-related, anthropometric and biochemical measurements were obtained from the medical records.

#### Data analysis

Data analysis was performed using the Statistical Package for the Social Science version 18.0 for Windows (SPSS Inc., Chicago, IL, USA). The prevalence of MetS and its components, and the awareness and knowledge of MetS among cancer survivors were examined using descriptive statistics, such as frequency and percentages, whereas the differences in the components of MetS, according to sex, were analyzed using the chi-square test. For all tests, *p*<0.05 was used to determine statistical significance.

## Results

### Demographic and cancer-related characteristics

Table 1 shows the demographic and cancer-related characteristics of the participants. A sample of 88 participants (72.0% men and 28.0% women), with a mean age of 66.7 years, participated in this study. The majority of the participants were married (83.0%), not engaged in any economic activity (92.0%) and of low economic status (70.5%). Participant distribution according to educational status was as follows: completion of elementary school, 17.0%; middle school, 28.4%; high school, 34.1%; and college, 13.6%. Moreover, 54.5% of participants had no religion, while 45.5% of participants reported a religious belief. Overall, 27.3% of participants were non-smokers, 52.3% were ex-smokers, and 20.5% were current smokers. With regard to monthly alcohol consumption, over half of the participants reported no alcohol consumption (78.4%). The distribution of cancer according to type was as follows: lung, 14.8%; stomach, 14.8%; colon/rectal, 18.2%; breast, 10.2%; hematologic malignancy, 23.9%; and other, 18.2%. For the majority of participants (73.9%) the time from cancer diagnosis was >1 year and <5 years and 53.4% of patients had a stage IV cancer (Table 1).

**Table 1:** Demographic and cancer-related characteristics.

Variables	Classification	Total (N = 88)	Male (n = 63)	Female (n = 25)
		N (%), mean±SD	n (%), mean±SD	n (%), mean±SD
Age (years)		66.66±9.57	66.43±9.16	67.24±10.73
Marital status	Single	12 (13.6)	9 (14.3)	3 (12.0)
	Married	73 (83.0)	52 (82.5)	21 (84.0)
	Other	3 (3.4)	2 (3.2)	1 (4.0)
Religion	None	48 (54.5)	32 (50.8)	8 (32.0)
	Yes	40 (45.5)	31 (49.2)	17 (68.0)
Educational status	Less than or equivalent to elementary school	21 (17.0)	11 (17.5)	10 (40.0)
	Middle school	25 (28.4)	17 (27.0)	8 (32.0)
	High school	30 (34.1)	27 (42.9)	3 (12.0)
	Less than or equivalent to college	12 (13.6)	8 (12.7)	4 (16.0)
Economic activity	None	81 (92.0)	56 (88.9)	25 (100.0)
	Yes	7 (8.0)	7 (11.1)	0 (0.0)
Economic status	High	1 (1.1)	1 (1.6)	0 (0.0)
	Moderate	25 (28.4)	17 (27.0)	8 (32.0)
	Low	62 (70.5)	45 (71.4)	17 (68.0)
Monthly alcohol consumption	None	69 (78.4)	44 (69.8)	25 (100.0)
	Present	19 (21.6)	19 (30.2)	0 (0.0)
Smoking	Non-smoker	24 (27.3)	5 (7.9)	19 (76.0)
	Ex-smoker	46 (52.3)	40 (63.5)	6 (24.0)
	Current smoker	18 (20.5)	18 (28.6)	0 (0.0)
Cancer type	Lung	13 (14.8)	10 (15.9)	3 (12.0)
	Stomach	13 (14.8)	11 (17.5)	2 (8.0)
	Colon/rectal	16 (18.2)	14 (22.2)	2 (8.0)
	Breast	9 (10.2)	0 (0.0)	9 (36.0)
	Hematologic malignancy	21 (23.9)	14 (22.2)	7 (28.0)
	Other	16 (18.2)	14 (22.2)	2 (8.0)
Cancer duration	<1 year	12 (13.6)	11 (17.5)	1 (8.3)
	≥ 1 year and <5 years	65 (73.9)	45 (71.4)	20 (30.8)
	≥5 years	11 (12.5)	7 (11.1)	4 (36.4)
Tumor stage	I	6 (6.8)	3 (4.8)	3 (12.0)
	II	16 (18.2)	10 (15.9)	6 (24.0)
	III	15 (17.0)	10 (15.9)	5 (20.0)
	IV	47 (53.4)	37 (58.7)	10 (40.0)
	Unknown	4 (4.5)	3 (4.8)	1 (4.0)

SD: standard deviation

### Prevalence of MetS and its components among cancer survivors

Table 2 shows the prevalence of MetS and its components among cancer survivors who met the NCEP criteria [6]. The proportion of cancer survivors who met each MetS criterion was as follows: WC >90 cm in men and >80 cm in women, 33.0%; triglyceride levels ≥150 mg/dL or medication use, 20.5%; HDL cholesterol levels <40 mg/dL in men and <50 mg/dL in women or medication use, 28.4%; BP ≥130/85 mmHg or antihypertensive medication use, 40.9%; and FBS levels ≥100 mg/dL or medication use (insulin or oral agents), 77.3%. Overall, 34.1% of the participants had MetS. Overall, 20.5% of the participants met the criteria for triglycerides (≥150 mg/dL or medication use), including 14.3% of men and 36.0% of women (the difference between the sexes was significant, *p* = 0.023). Moreover, 28.4% of the participants met the criteria for HDL (<40 mg/dL or medication use), including 20.6% of men and 48.0% of women (*p* = 0.010) (Table 2).

**Table 2:** Prevalence of MetS and its components among cancer survivors.

Variables	Total, N (%)	Gender		
		Male, n (%)	Female, n (%)	*p*
Metabolic syndrome	30 (34.1)	17 (27.0)	13 (52.0)	0.026
WC >90 cm in men and >80 cm in women	29 (33.0)	17 (27.0)	12 (48.0)	0.059
Triglyceride levels 80 mg/dL or medication use	18 (20.5)	9 (14.3)	9 (36.0)	0.023
HDL cholesterol levels <40 mg/dL in men and <50 mg/dL in women or medication use	25 (28.4)	13 (20.6)	12 (48.0)	0.010
BP ≥130/85 mmHg or antihypertensive medication use	36 (40.9)	22 (34.9)	14 (56.0)	0.070
FBS levels 100 mg/dL or medication use (insulin or oral agents)	68 (77.3)	49 (71.6)	19 (76.0)	0.858

WC: waist circumference, TG: triglyceride, HDL: high-density lipoprotein, HTN: hypertension, FBS: fasting blood sugar

### Awareness of MetS and its components among cancer survivors

Table 3 describes the awareness of MetS and its components among cancer survivors. Only 6.8% of participants were actually diagnosed with MetS. Over half of the participants had heard of MetS (56.8%). The extent of participant knowledge of MetS among the cancer survivors was distributed as follows: very much, 8.0%; somewhat, 44.3%; not much, 46.6%; and not at all, 1.1%. Furthermore, 52.3% of participants wished to consult with their healthcare provider regarding MetS. Awareness of each component of MetS among participants was as follows: WC levels, 78.4%; FBS levels, 31.8%; BP, 83%; cholesterol levels, 4.5%; and triglyceride levels, 2.3% (Table 3).

**Table 3:** Awareness of MetS and its components among cancer survivors.

Variables	Classification	n (%)
MetS diagnosis	Yes	6 (6.8)
	No	82 (93.2)
Ever heard about MetS	Yes	50 (56.8)
	No	38 (43.2)
Want to know about MetS	Very much	7 (8.0)
	Somewhat	39 (44.3)
	Not much	41 (46.6)
	Not at all	1 (1.1)
Want to consult with a healthcare provider regarding MetS	Yes	46 (52.3)
	No	42 (47.7)
Awareness of WC	Yes	69 (78.4)
	No	19 (21.6)
Awareness of FBS level	Yes	28 (31.8)
	No	60 (68.2)
Awareness of BP	Yes	73 (83.0)
	No	15 (17.0)
Awareness of the cholesterol level	Yes	4 (4.5)
	No	84 (95.5)
Awareness of the triglyceride level	Yes	2 (2.3)
	No	86 (97.7)

MetS: metabolic syndrome, WC: waist circumference; FBS: fasting blood sugar; BP: blood pressure

### Knowledge level of MetS among cancer survivors

The proportion of correct answers to the questions assessing the knowledge of MetS were distributed as follows: 39.1% for diabetes-related questions, 47.2% for adiposity-related questions, 46.8% for hypertension-related questions, 36.7% for cholesterol level-related questions, 45.5% for arteriosclerosis-related questions, 37.8% for myocardial infarction-related questions, and 62.8% for stroke-related questions. Therefore, the highest proportion of correct answers was obtained for questions regarding stroke, and the lowest was obtained for questions regarding cholesterol levels (Table 4).

**Table 4:** Knowledge level of MetS among cancer survivors.

Component	Maximum score	Mean±SD	Average percentage of correct answers	SD
Diabetes	12	5.07±3.04	39.1	16.5
Adiposity	9	4.25±2.26	47.2	17.2
Hypertension	10	4.68±2.06	46.8	15.7
Cholesterol level	6	2.20±1.43	36.7	24.9
Arteriosclerosis	16	7.73±4.60	45.5	16.1
Myocardial infarction	10	5.67±3.69	37.8	17.5
Stroke	12	6.28±2.69	62.8	22.4

SD: standard deviation

### Proportion of correct answers for the MetS components among cancer survivors

Table 5 shows the proportion of correct answers concerning knowledge of the MetS components (diabetes, obesity, hypertension, high serum cholesterol level, arteriosclerosis, stroke, and myocardial infarction) among the cancer survivors. With regards to the diabetes-related questions, the highest proportion of correct answers was obtained for the questions concerning eye complications and overall, 75.0% of the participants correctly identified diabetes complications. The question with the lowest proportion of correct answers regarded the statement that excess sugar entered the cells in diabetes; only 11.4% of the participants correctly identified this statement as false. Among the obesity-related questions, the question related to the effects of adiposity was associated with the highest proportion of correct answers (75.0%). Conversely, only 22.7% of the participants correctly identified the statement that in diabetes, sugar could not move in the blood. With regards to the hypertension-related questions, the highest proportion of correct answers (71.6%) was achieved for the question on the heredity of hypertension. In contrast, the question with the lowest proportion of correct answers (17.0%) included the statement that hypertension can be caused by disorders of the thyroid gland. Among the serum cholesterol level-related questions, the highest proportion of correct answers (64.8%) concerned the statement on medication. Conversely, the question with the lowest proportion of correct answers (4.5%) included the statement that fatigue is a frequent symptom of high serum cholesterol levels. Among the arteriosclerosis-related questions, the highest proportion of correct answers (73.9%) related to the statement that arteriosclerosis increases the risk of suffering a stroke, while the question with the lowest proportion of correct answers (2.3%) included the statement that arteries contract with arteriosclerosis. Among the stroke-related questions, the highest proportion of correct answers (87.5%) included the statement that stroke affects the brain, while the question with the lowest proportion of correct answers (11.4%) included the statement that stroke is frequently preceded by chest pain. Among the questions on myocardial infarction, the highest proportion of correct answers (63.6%) regarded the statement that smoking is a minor risk factor for myocardial infarction. Conversely, the lowest proportion of correct answers (5.7%) included the statement that myocardial infarction is usually preceded by loss of sensation and numbness (Table 5).

**Table 5:** Proportion of correct answers regarding the MetS components among cancer survivors.

Components	Question	Correct answer	n	%
Diabetes	There are several different types of diabetes.	True	35	39.8
	Hereditary factors play a major role in the development of diabetes.	True	49	55.7
	Hereditary factors only play a minor role in the development of diabetes.	False	42	47.7
	Eye disorders can be a consequence of diabetes.	True	66	75.0
	For some individuals with diabetes, it is not advisable to take insulin.	True	29	33.0
	Individuals with diabetes may only eat special types of sweets.	False	36	40.9
	In diabetes, sugar cannot enter the cells sufficiently.	True	18	20.5
	Poor appetite is a frequent symptom of diabetes.	False	28	31.8
	In diabetes, too much sugar enters the cells.	False	10	11.4
	Frequent urination is a classic symptom of diabetes.	True	51	58.0
	Individuals with diabetes must receive insulin shots.	False	27	30.7
	Arteriosclerosis is one of the sequelae of diabetes.	True	36	40.9
	In diabetes, sugar cannot move in the blood.	False	20	22.7
Obesity	Obese individuals have an elevated risk of suffering a myocardial infarction.	True	64	72.7
	Adiposity is not only caused by nutrition, but other factors contribute as well.	True	66	75.0
	An excessively fatty, high-caloric diet is the only factor that determines adiposity.	False	35	39.8
	The terms ‘overweight’ and ‘adiposity’ are synonyms.	False	42	47.7
	Cessation of breathing while sleeping is a possible consequence of adiposity.	True	36	40.9
	Obese individuals have the same risk as non-adipose individuals of suffering a stroke.	False	52	59.1
	Obese individuals are more likely to suffer from arteriosclerosis.	True	27	30.7
	Liposuction is the best possible treatment for increased adiposity.	False	32	36.4
	Adiposity can be treated surgically.	True	20	22.7
Hypertension	Hypertension is associated with heredity.	True	63	71.6
	For the most part, a single concrete reason of why a patient suffers from hypertension can be determined.	False	40	45.5
	After medication has reduced the hypertension, the medication can usually be discontinued.	False	43	48.9
	People with hypertension are as likely to suffer from arteriosclerosis as those with normal blood pressure.	False	33	37.5
	Individuals with hypertension are less likely to suffer from arteriosclerosis.	False	40	45.5
	Hypertension can cause dizziness.	True	61	69.3
	Hypertension can be caused by disorders of the thyroid gland.	True	15	17.0
	Hypertension can cause renal damage.	True	38	43.2
	Hypertension can lead to eye disorders.	True	51	58.0
	Hypertension can be caused by cerebral tumors.	True	28	31.8
High serum cholesterol	A low cholesterol diet can supplement therapy for high serum cholesterol levels.	True	50	56.8
	High serum cholesterol levels can be treated with medication.	True	57	64.8
	High serum cholesterol levels do not cause acute ailments.	True	7	8.0
	High serum cholesterol levels are not associated with hereditary factors.	False	23	26.1
	High serum cholesterol levels promote arteriosclerosis.	True	53	60.2
	Fatigue is a frequent symptom of high serum cholesterol levels.	False	4	4.5
Arteriosclerosis	Arteriosclerosis increases the risk of suffering a stroke.	True	65	73.9
	Leg pain is a symptom of arteriosclerosis.	True	39	44.3
	In arteriosclerosis, arteries become softer.	False	43	48.9
	Arteriosclerosis can be cured completely.	False	23	26.1
	In arteriosclerosis, arteries contract.	False	2	2.3
	In arteriosclerosis, arteries become less elastic.	True	44	50.0
	As a result of arteriosclerosis, blood pressure is likely to decline.	False	43	48.9
	As a result of arteriosclerosis, blood pressure is likely to increase.	True	48	54.5
	High blood pressure and arteriosclerosis are not linked with each other.	False	45	51.1
	In arteriosclerosis, a sustainer can be inserted into the artery in order to stabilize it.	True	29	33.0
	The risk of developing arteriosclerosis is not hereditary.	False	27	30.7
	Arteriosclerosis can cause renal damage.	True	44	50.0
	In arteriosclerosis, blood platelets accumulate on the arterial walls.	True	41	46.6
	In arteriosclerosis, fat accumulates on the arterial walls.	True	51	58.0
	Individuals with high blood pressure are more likely to suffer from arteriosclerosis.	True	58	65.9
	Medication can completely remove sediments from the arteries.	False	30	34.1
	In arteriosclerosis, arteries become brittle.	True	49	55.7
Stroke	A stroke affects the brain.	True	77	87.5
	If a patient survives a stroke, there are usually no permanent consequences.	False	56	63.6
	Permanent speech defects are possible consequences of a stroke.	True	75	85.2
	A stroke is often followed by memory dysfunction.	True	73	83.0
	There are different types of stroke.	True	53	60.2
	A stroke is caused by arterial occlusion.	True	58	65.9
	The nutrient supply to the brain is not affected by a stroke.	False	43	48.9
	A stroke is frequently preceded by chest pain.	False	10	11.4
	A stroke is frequently preceded by speech problems.	True	70	79.5
	Individuals with diabetes are more likely to suffer a stroke.	True	38	43.2
Myocardial infarction	Smoking is a minor risk factor with respect to myocardial infarction.	False	56	63.6
	When suffering a myocardial infarction, pain may radiate to the arms.	True	35	39.8
	The oxygen supply to the heart is not affected by a myocardial infarction.	False	53	60.2
	Hereditary factors play a role in the risk of suffering a myocardial infarction.	True	36	40.9
	After myocardial infarction, anticoagulants are administered.	True	34	38.6
	A myocardial infarction is often preceded by shortness of breath.	True	49	55.7
	A myocardial infarction is caused by arterial obstruction.	True	54	61.4
	Damage caused by a myocardial infarction is not usually permanent.	False	22	25.0
	After a myocardial infarction, parts of cardiac muscle tissue can die.	True	40	45.5
	A myocardial infarction must be treated surgically.	False	14	15.9
	In myocardial infarction, cardiac muscle tissue dies.	True	10	11.4
	Diabetes is a predisposing factor for myocardial infarction.	True	31	35.2
	When suffering a myocardial infarction, pain may radiate to the stomach.	True	25	28.4
	A myocardial infarction is usually preceded by loss of sensation and numbness.	False	5	5.7
	A myocardial infarction can manifest itself through nausea and vomiting.	True	35	39.8

## Discussion

In this study, the prevalence of MetS among cancer survivors was 34.1% which is consistent with the findings of a previous study [19]. However, there were significant differences in the reported prevalence of MetS among cancer patients in another previous study [20], which reported the differences in the prevalence of MetS among women with breast cancer according to tumor stage. Therefore further research involving large participant samples are required. Although the prevalence of MetS among participants was 34.1%, only 6.8% of these had a diagnosis of MetS. Based on our review, this finding may reflect a lack of interest and awareness of MetS among cancer survivors. We hypothesized that the patients did not recognize the seriousness of chronic diseases like MetS as they were primarily concerned with the risk of cancer recurrence. It is possible that cancer survivors, as well as medical professionals, may predominantly focus on cancer recurrence and diagnose individual diseases rather than adopting an integrated approach and diagnosing MetS. Therefore, it is important that patients and healthcare providers focus not only on cancer recurrence but also on health education for managing the risk factors of MetS among cancer survivors.

In this study, approximately 56.8% of cancer survivors were aware of MetS. In fact, over half of the participants reported that they had heard of MetS. The awareness rate of MetS in the present study is greater than that reported in a previous study targeting elderly patients (9.0%) [18]. Evidence indicates that participants learn about MetS from acquaintances, the media, physicians, or others [21], and cancer survivors were more likely to be concerned about the disease than elderly subjects. A more accurate interpretation of the condition can be made as a result of the higher awareness of MetS, compared to the previous 10 years. In a previous study [18], over half of the participants were not aware of their triglyceride levels (94.4%), cholesterol levels (87.8%), or FBS levels (65.9%). These findings are consistent with the findings of this study in which a considerable proportion of participants were not aware of their triglyceride levels (97.7%), cholesterol levels (95.5%), and FBS levels (68.2%). These results suggest that the level of awareness regarding diabetes and lipid abnormalities among cancer survivors was lower than that of hypertension and obesity. Hence, it is crucial to emphasize the importance of the long-term self-management of blood glucose or lipid levels. Moreover, the present study revealed only minimal knowledge of and reduced perceived awareness of MetS, although 52.3% of the respondents reported that they would like to receive counseling if they had MetS. We postulate that the participation rate in health improvement programs will be high among cancer survivors and that these strategies can achieve positive results. Therefore, active interventions, such as education and public awareness campaigns, including mass media that promote health-related information [22], are needed to enhance the knowledge and awareness of MetS among cancer survivors.

Overall, these results indicate that cancer survivors were most knowledgeable about stroke and least knowledgeable about cholesterol levels, consistent with a previous report investigating the knowledge of MetS among college students [23]. Overall, the proportion of correct answers in this study was lower than that reported in the previous studies [18,23], which may be due to the increased age and lower socioeconomic statuses of participants in our sample.

With regards to knowledge about diabetes, 59.1% of the participants believed that patients with diabetes should only eat special types of sweets, which may reflect the public opinion regarding diabetes. These results indicate that the understanding of the etiology and treatment of diabetes is poor. Patients with diabetes also have greater risk of developing cancer than non-diabetic patients [24]. Therefore, interventions to enhance the awareness and knowledge of MetS among cancer survivors should prioritize diabetes education. These efforts will help to reduce the risk of subsequently developing MetS.

Moreover, adiposity is a risk factor for MetS. Over half of the participants in this study were aware of the cause of adiposity, and that it was a risk factor for MetS (72.7%) and stroke (59.1%). However, over half of the participants were not aware that the terms ‘overweight’ and ‘adiposity’ are synonyms (52.3%), with the former indicating a gain in body weight and the latter indicating an excess of body fat. Furthermore, 63.6% of the participants believed that liposuction was the best treatment for adiposity. Confusion regarding overweight and adiposity and the false belief that liposuction is the best treatment for adiposity may prevent patients from leading healthy lifestyles. Hence, it is important to enhance the awareness and knowledge of obesity among cancer survivors.

With regards to the hypertension-related questions, over half of the participants were aware that hypertension can be hereditary and causes dizziness. However, hypertension is referred to as a “silent” killer because it usually develops without any symptoms or warning signs, progressing before individuals realize they are hypertensive. Approximately 1 in 5 (20.4%) adults in the United States are estimated to have high BP, but are unaware of this [25,26].

The questions on high serum cholesterol levels were associated with the lowest proportion of correct answers. Over half of the participants (64.8%) believed that high serum cholesterol levels could be treated with medication. The idea that hypercholesterolemia can only be treated with medication may lead to patients neglecting exercise, healthy eating habits, and weight management. Furthermore, approximately half of the participants were not aware that elevated cholesterol levels could lead to thickening of the wall of the arteries, subsequently leading to stiffness and loss of elasticity. Therefore, the majority of the participants were not aware of the association between cholesterol levels and arteriosclerosis. Furthermore, 73.9% of the participants held the false belief that arteriosclerosis could be completely cured. These false beliefs may lead to the lack of recognition of the importance of maintaining a healthy lifestyle.

The questions related to stroke were associated with the highest proportion of correct answers. In fact, 87.5% of the participants were aware that a stroke affects the brain; 88.6% of participants believed that stroke is frequently preceded by chest pain, which is consistent with the findings of a study conducted by Becker et al. [18] involving students. The authors concluded that over one-third of the participants believed that stroke often begins with chest pain.

With regard to the myocardial infarction-related questions, 63.6% of the participants believed that smoking is a risk factor for myocardial infarction. Specifically, 75.0% of the participants were aware that the damage caused by myocardial infarction was not generally permanent. Moreover, 84.1% of the participants were aware that myocardial infarction should be treated surgically. As cardiovascular disease can cause permanent damage to cardiac tissue, patients should be educated about the early risk factors for heart disease.

Interestingly, communication with healthcare providers has been reported as the preferred source of information [27]. However, most cancer survivors were more focused on their diagnosis and information about treatment, side-effects, and ways to manage cancer itself [28]. Not surprisingly, healthcare providers are also more focused on cancer follow-up and diagnostic examination of recurrent cancers [28]. For these reasons, although patients, families, and healthcare providers often find it difficult to discuss issues regarding MetS, healthcare providers have to be responsible for providing educational support to families as well as cancer survivors in order to create the necessary awareness about MetS while undergoing cancer treatment [29]. Ultimately, healthcare providers should provide total care to cancer survivors to manage MetS as well as cancer symptoms and recurrence. It is important that healthcare providers are available, attentive, and sensitive to these concerns [29].

Therefore, it is vital that nurses are involved in tailoring information to the needs of individual patients. Nurses can better respond to patient requirements by assessing the information needed by the patients and clarifying their doubts [30]. Furthermore, nurses can improve patient satisfaction by evaluating their understanding of the information and attempting to resolve any confusion [31,32]. Previous studies indicated that interventions promoting healthy behaviors, including regular physical activity, weight management, and a healthy diet, can reduce the prevalence of MetS [26,27,32,33,34]. Increasing the level of knowledge of the risk factors of MetS among patients can help to enhance the lifestyles of cancer survivors. Lifestyle changes are an effective strategy for reducing the incidence of MetS [35]. Based on these findings, guidelines for an educational intervention aimed at cancer survivors with a high risk of MetS can be developed.

The present study has a number of limitations. First, the study comprised a small sample size, which limits the generalizability of these findings and the statistical power to detect significant differences between genders. Therefore, the results of this study should be interpreted with caution, as they may not be generalizable to all cancer survivors. Another limitation is the cross-sectional design, which cannot distinguish whether the incorrect answers were due to the lack of awareness among cancer survivors or incorrect pre-existing knowledge. Finally, limited participant understanding, as reflected by the low socioeconomic statuses of the participants in our sample, may also have affected the results. Despite these limitations, in our view, the findings of this study can be used to develop educational interventions aimed at addressing the needs of cancer survivors.

## Conclusion

In conclusion, our results suggest that level of awareness and knowledge of cancer survivors regarding the components of metabolic syndrome is poor; it is essential to assess cancer survivors’ awareness and knowledge to develop educational strategies and to evaluate the influences of these strategies on the compliance and quality of life to improve survivorship among cancer survivors.
